# Investigation of simultaneously existed Raman scattering enhancement and inhibiting fluorescence using surface modified gold nanostars as SERS probes

**DOI:** 10.1038/s41598-017-07311-8

**Published:** 2017-07-28

**Authors:** Feng Shan, Xiao-Yang Zhang, Xing-Chang Fu, Li-Jiang Zhang, Dan Su, Shan-Jiang Wang, Jing-Yuan Wu, Tong Zhang

**Affiliations:** 1 Joint International Research Laboratory of Information Display and Visualization, School of Electronic Science and Engineering, Southeast University, Nanjing, 210096 People’s Republic of China; 2 Key Laboratory of Micro-Inertial Instrument and Advanced Navigation Technology, Ministry of Education, and School of Instrument Science and Engineering, Southeast University, Nanjing, 210096 People’s Republic of China; 3 Suzhou Key Laboratory of Metal Nano-Optoelectronic Technology, Suzhou Research Institute of Southeast University, Suzhou, 215123 People’s Republic of China

## Abstract

One of the main challenges for highly sensitive surface-enhanced Raman scattering (SERS) detection is the noise interference of fluorescence signals arising from the analyte molecules. Here we used three types of gold nanostars (GNSs) SERS probes treated by different surface modification methods to reveal the simultaneously existed Raman scattering enhancement and inhibiting fluorescence behaviors during the SERS detection process. As the distance between the metal nanostructures and the analyte molecules can be well controlled by these three surface modification methods, we demonstrated that the fluorescence signals can be either quenched or enhanced during the detection. We found that fluorescence quenching will occur when analyte molecules are closely contacted to the surface of GNSs, leading to a ~100 fold enhancement of the SERS sensitivity. An optimized Raman signal detection limit, as low as the level of 10^−11^ M, were achieved when Rhodamine 6 G were used as the analyte. The presented fluorescence-free GNSs SERS substrates with plentiful hot spots and controllable surface plasmon resonance wavelengths, fabricated using a cost-effective self-assembling method, can be very competitive candidates for high-sensitive SERS applications.

## Introduction

Surface-enhanced Raman scattering (SERS) active substrate is currently a hot topic in highly sensitive optical spectral sensing applications because of its short time-consumption and reproducibility^1–4^. Uniformly distributed metal nanostructures with abundant hot spots supporting localized surface plasmon resonance (LSPR) are the critical elements for high-sensitivity SERS active substrates^5, 6^. The hot spots mainly at the sharp tips of metal nanostructures produce a strongly localized electromagnetic field enhancement, which is beneficial for SERS signal enhancement. Therefore, metal nanostructures with densely packed sharp tips, such as nanostars^7–10^ and nanoplates^11–15^ are the best promising candidates for high-performance SERS probes.

To obtain such nanostructures with well controlled morphology and size for SERS application, chemical synthesis methods were usually used^4, 9, 12, 16^. However, such chemically synthesized metal nanostructures containing organic shells (capping agents, or modifier)^17^ always generate serious background fluorescence noise which limits the SERS detection sensitivity greatly. Although this phenomenon has already been observed in many SERS detection experiments^18–20^, few methods were proposed to solve this problem. The only proposed strategy to avoid the influence of fluorescence is changing the excitation wavelength for the Raman measurement to longer wavelengths^21–23^. However, when excitation wavelength with lower photon energy was used, the SERS enhancement factor (EF) would also be decreased obviously. Therefore, SERS detection strategies which could effectively inhibit the fluorescence noise without decreasing, or even increasing SERS EF are highly expected.

In this paper, we focused on the performance improvement of SERS substrates self-assembled by chemical synthesized gold nanostars (GNSs). We investigated the phenomenon that the Raman scattering enhancement and inhibiting fluorescence during SERS detection process simultaneously existed, which greatly influenced the SERS EF. Based on plasmon-induced surface resonance energy transfer theory, we precisely controlled the space between the metal and organic molecules by surface modification of GNSs to inhibit the fluorescence in SERS measurement, rather than simply changing the excitation wavelength. Once the fluorescence was inhibited, we obtained sensitive GNSs SERS probes with optimum sensitivity 10^2^ times higher than the original ones which is a very competitive result for high-sensitivity SERS detection. Meanwhile, we showed various technologies including precise control of GNSs morphology, large-scale and uniform preparation of GNSs substrates and several surface modification methods for molecules distance control, which can be widely applied in the fields of optical sensing, processing and display.

## Results and Discussions

### High-yield GNSs characterization

**Figure 1:**
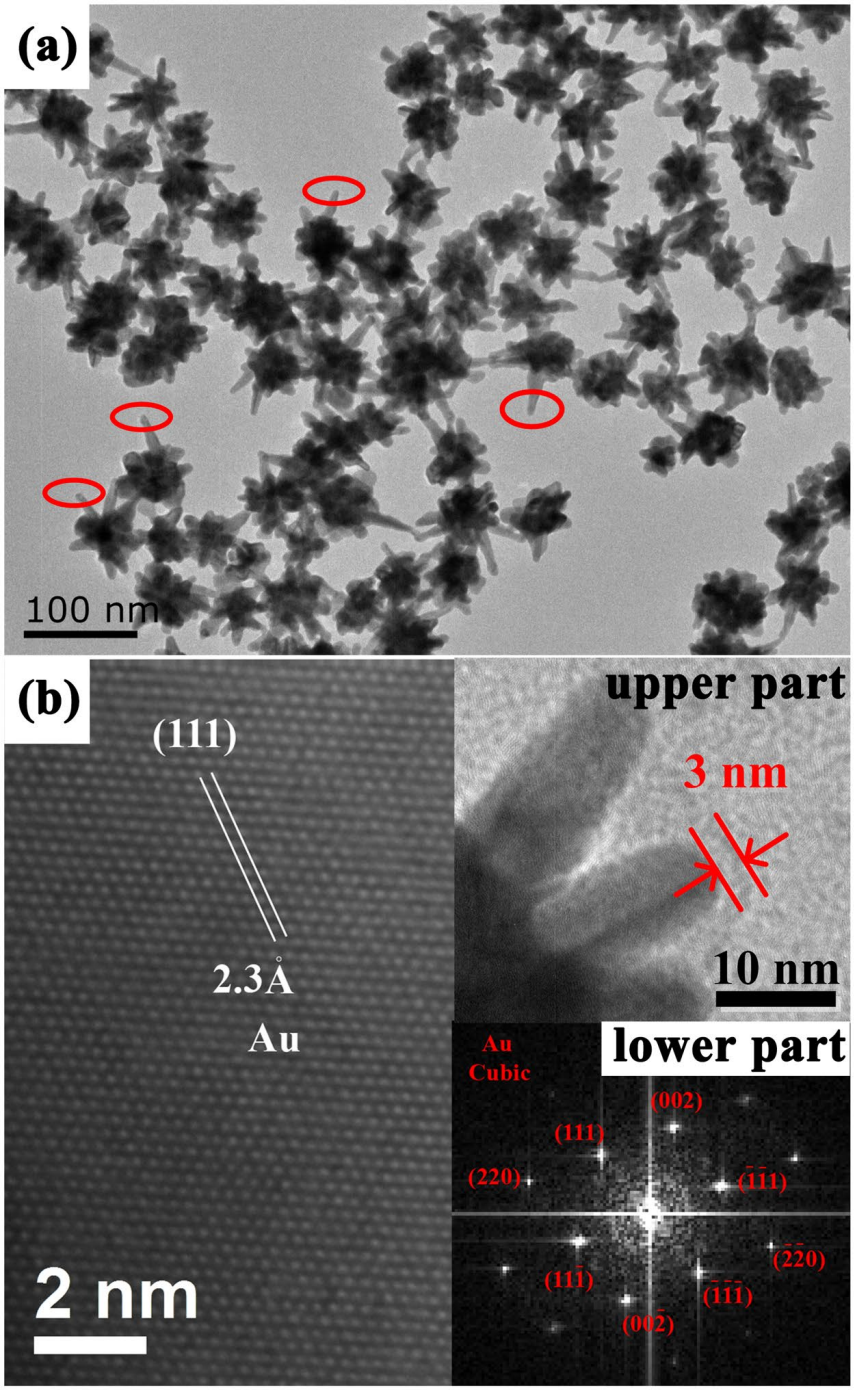
(**a**) TEM image of GNSs with AgNO_3_ concentration of 30 mM. (**b**) HRTEM image of GNS. The inset of bottom is the corresponding FFT pattern of the GNS.

First, we chemically synthesized GNSs with well-controlled morphology as SERS probes combined from previously reported methods^24–31^. High-yield (~100%) GNSs samples with different length of tips were obtained as shown in Fig. 1a. Each GNS was comprised of a quasi-sphere core (~50 nm) and several sharp tips. We also observed there was organic shell on the surface of GNSs (the upper part of Fig. 1b). The thickness of organic shell was about 3 nm. It is interesting that the length of tips on GNSs can be well-controlled by adjusting AgNO_3_ concentration. Therefore, the hot spots mainly located on the tip (red circle in Fig. 1a) increased with the increased concentration of AgNO_3_. The crystalline structure of the GNSs can be identified from the high-resolution TEM (HRTEM) image of the GNSs in Fig. 1b. The spacing between adjacent lattice planes is 0.23 nm. It corresponds to the <111> lattice plane of face-centered cubic (fcc) Au^28–30^. The fast Fourier transform (FFT) patterns obtained from the above HRTEM image (the lower part of Fig. 1b) shows a hexagonal symmetry diffraction pattern, which indicates that the tips of GNSs are single-crystal with the growth direction of Au <111>. Therefore, the overall results demonstrate that the synthesized GNSs with high uniformity and single-crystal property facilitate the fabrication of reproducible SERS substrates.

**Figure 2:**
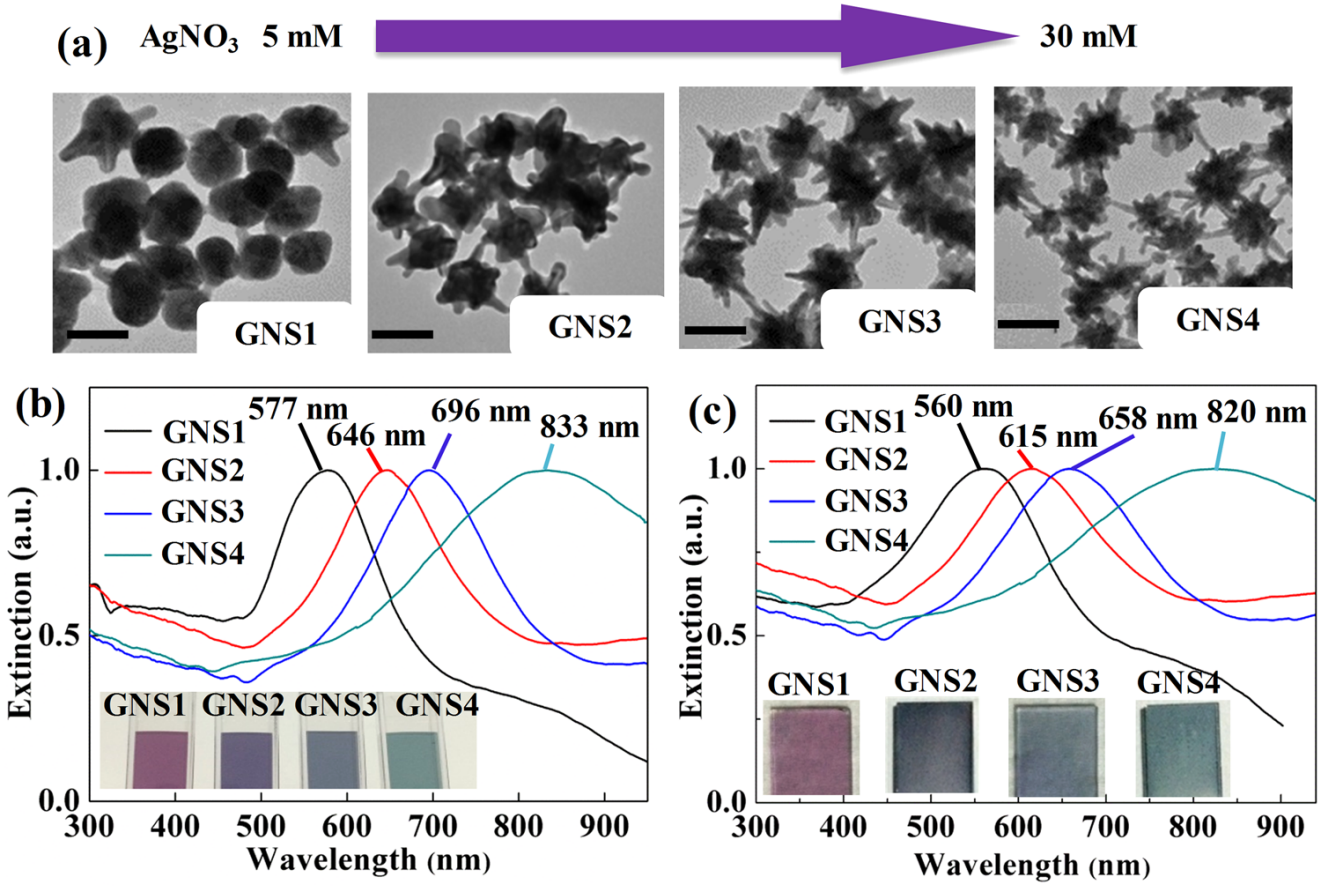
(**a**) Representative TEM images of GNSs synthesized with different AgNO_3_ concentrations (GNS1: 5 mM, GNS2: 10 mM, GNS3: 20 mM, GNS4: 30 mM). (**b**) Normalized extinction spectra of the GNSs solutions, (inset) solutions photographs and (**c**) GNSs films correspond to the above four samples, (inset) films photographs.

Figure 2a shows the relationship between the morphologies and concentrations of AgNO_3_. Four samples, named GNS1 to GNS4, were synthesized to show the important role of hot spots for SERS. Meanwhile, with the increase length of tips, as well as the improvement of the anisotropy of GNSs, the color of the GNSs solutions changed from purple to blue as shown in the inset of Fig. 2b. Accordingly, the spectral measurement (Fig. 2b) indicates the LSPR bands^11, 25^ of the GNSs solution red-shifted, covering the entire Vis-NIR region. It provides various choices for SERS probing in both the visible and NIR diagnostic windows^28^.

### Preparation and characterization of SERS substrates

**Figure 3:**
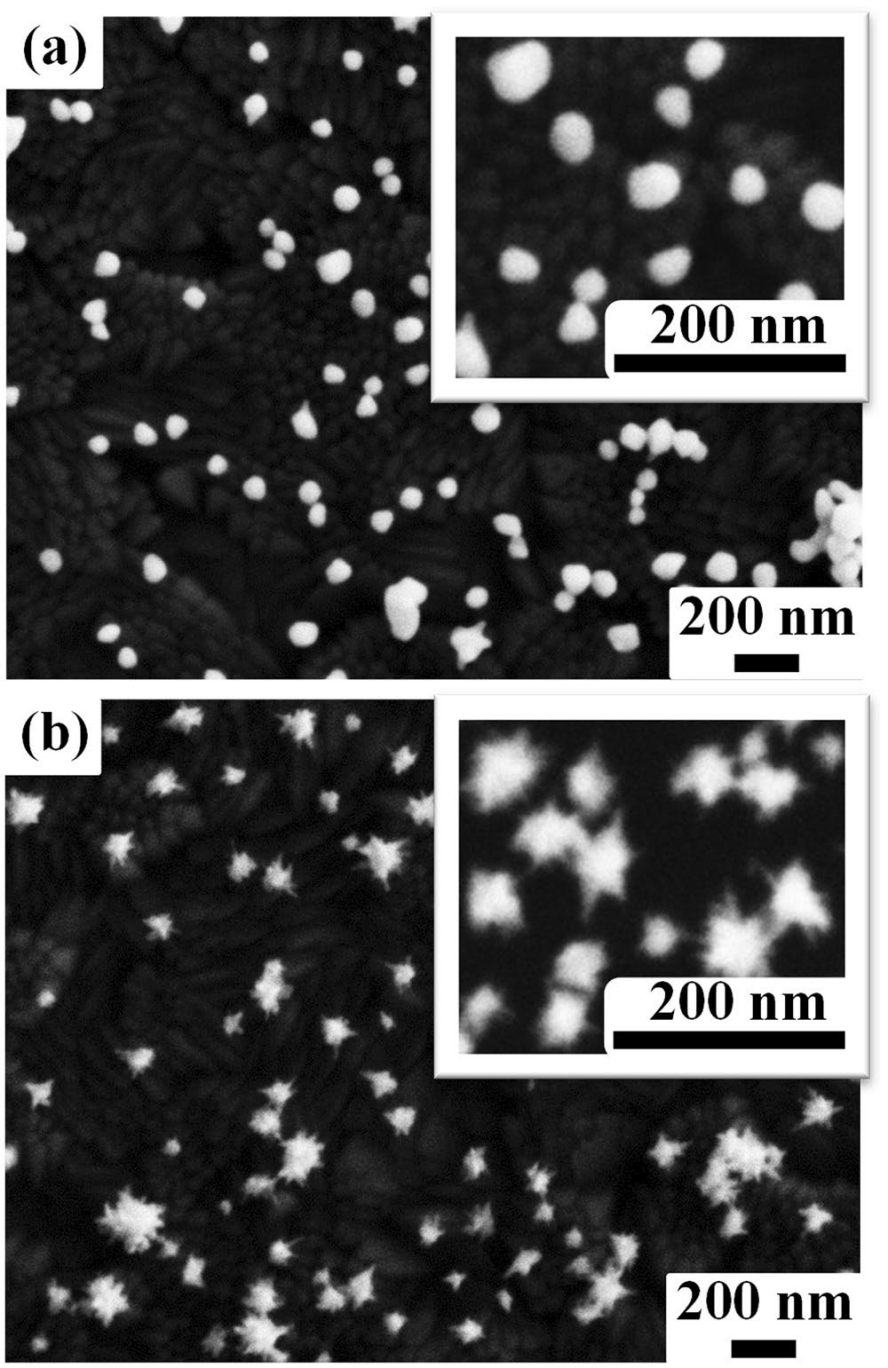
SEM images of the GNSs film corresponding to samples GNS1 (**a**) and GNS4 (**b**), respectively. Insets in (**a**) and (**b**) correspond to higher magnification SEM images for samples GNS1 and GNS4, respectively.

A simple and effective assistance-free self-assembly process was used to fabricate SERS substrates with large-scale monolayer and uniformly distributed GNSs. ITO glass slabs with a size of 1 × 3 cm were washed by using ultrapure water first. Then, the cleaned ITO glass slabs were immersed in GNSs solutions for 12 h. The GNSs solutions were used without any post-processing after producing by ‘GNSs synthesis’ section method. We also compared the effect of the immersion time on the distribution of GNSs on glass slabs. It was found that the GNSs’ distribution became stable and did not change obviously after 12 h even when they were immersed more time. The corresponding extinction spectra of films from GNS1 to GNS4 are shown in Fig. 2c. The extinction spectra of the four films were all broadening compared with that of the corresponding solutions. Meanwhile, the resonance band of films had a blue shift of dozens of nanometers. This phenomenon can be attributed to the variation of surrounding environment and the aggregation of GNSs on the substrates^11, 32^. The insets of Fig. 2c showed the photographs of GNSs films, indicating the uniformity of GNSs films in large scale. The SEM images of samples GNS1 and GNS4 were shown in Fig. 3a and b, respectively. The insets of these two figures clearly showed the morphological differences between these two samples. Note that, as the densities of the two films are on the same order of magnitude, the SERS EF is mainly influenced by the morphology, i.e. the length of tips of GNSs.

### SERS measurements of GNSs SERS substrates

**Figure 4:**
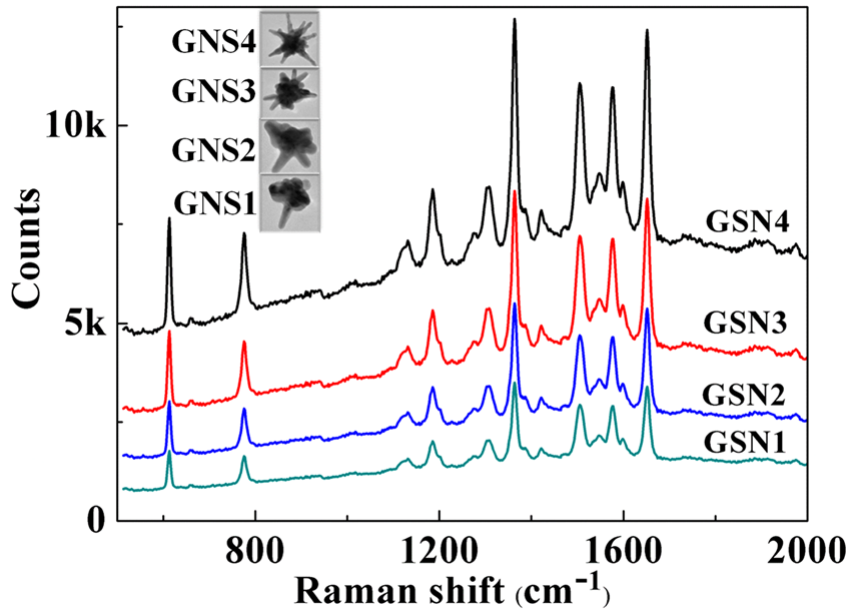
SERS spectra of R6G at a concentration of 1 × 10^−7^ M using SERS probes from GNS1 to GNS4, respectively. The laser wavelength is 514 nm.

Next, we investigated the SERS performance of these four GNSs substrates with a series of experimental comparisons. First, we verified the importance of the tips. Figure 4 showed the SERS spectra of GNSs SERS probes (GNS1 to GNS4) when Rhodamine 6G (R6G) molecules with a concentration of 10^−7^ M were employed as the analyte. Typical Raman characteristic peaks of R6G are clearly shown in these curves which are well matched with the results shown in the published literature^33^. Meanwhile, it is clearly seen that the GNS4 SERS probes with the longest tip displayed the strongest SERS signal, owing to the well-known hot spots effect^34^. For electromagnetic enhancement of SERS, the enhancement factor is proportional to the fourth power of the localized light intensity confined on the surfaces of metal nanostructures^35^. It has been verified that a single hot spot induced electromagnetic enhancement factor can reach as high as 10^9^, which is 3 to 4 orders of magnitude higher than that of an isolated metal nanoparticle. Therefore, SERS enhancement factor of metal nanostructures with plenty of hot spots is much higher than that of conventional spherical nanoparticles. For GNS4, due to the morphology complexity and anisotropic tip distribution, the resonant wavelength of different hot spots in GNS were quite different and extended to a much wider wavelength range far away from the main LSPR peak corresponding to their different dipole moments. Although their LSPR band dependent on their macroscopical distribution of mean size had red-shifted to a wavelength longer than the excitation wavelength, it still enable the excitation of a lot of hot spots contributing to the SERS enhancement at the excitation wavelength and led to a SERS enhancement factor much higher than other samples short of tips.

**Figure 5:**
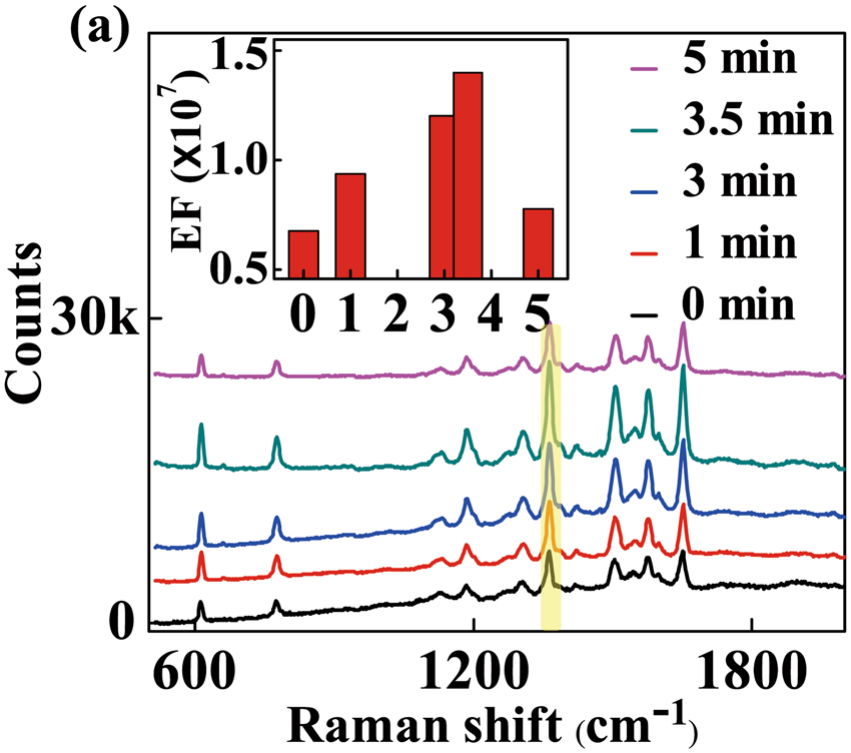
SERS spectra of R6G at a concentration of 1 × 10^−7^ M for GNS4 probe under different acetone treatment time.

Then, we focused on the inhibition of fluorescence noise during the SERS detection. As described above, in Fig. 1b, there are organic shells on the surface of GNSs, blocking the direct contact between the surface of metal and R6G molecules. We believe the existence of the organic shells will generate strong fluorescence noise, leading to the decrease of SERS signals. To verify this suspicion, we designed a series of experiments. We used acetone to dissolve the organic shells of GNSs by immersing the SERS substrates into acetone solution for different time. We put the glass slabs in a 50 mL beaker and then added acetone solvent quickly. We took the samples out of the beaker at different time (1 minute, 3 minutes, 3.5 minutes and 5 minutes). Finally, the samples were used as the SERS substrate in the next experiments. After the immersion of 10^−7^ M R6G, we compared the SERS performance of these samples, as shown in Fig. 5. The SERS EF was calculated by the reported method^36^. It is defined as EF = (*I*_*SERS*_ /*C*_*SERS*_)/(*I*_*0*_/*C*_*0*_), where *I*_*0*_ corresponds to the peak value of Raman signal obtained under ITO glass substrate at an R6G concentration of 10^−2^ M (Supplementary, Fig. S3). *I*_*SERS*_ corresponds to the peak value of Raman signal obtained under GNSs SERS substrate at an R6G concentration of 10^−7^ M. Here, the intensity at 1363 cm^−1^ Raman peak was selected as a reference value to compare the SERS EF, as illustrated in the inset of Fig. 5. When immersion time increased from 0 to 3.5 minutes, the SERS EF increased obviously. During this period, organic molecules on the surface of GNSs were dissolved, resulting in gradual decrease of the space between the metal and R6G molecules. To further verify this view, Energy-dispersive X-ray (EDX) spectrum was used to analyze the elements adsorbed onto the surface of GNSs before and after immersing in the acetone solution respectively (Supplementary Fig. S1). The results indicated that the organic molecules have been effectively dissolved after acetone immersion as we discussed above. With further increase of immersion time, the SERS EF decreased instead. It is because GNSs probes gradually decomposed without the protection of organic shells after long-time immersion. This can be directly seen from TEM image of GNS4 after acetone immersion of 5 minutes as shown in Fig. S2. The results exhibited that the sharp tips of GNS4 became much shorter or even disappeared. We also measured the extinction spectra of GNS4 with different immersion time in acetone as shown in Fig. S2(d). An obvious blue-shift of the maximum extinction wavelength can be observed from the comparison of these extinction spectra, which indicated the removal of organic shells and the decomposition of tips of GNS4 by acetone solvent.

**Figure 6:**
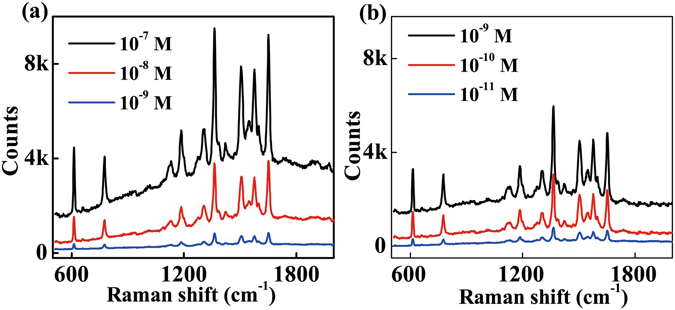
(**a**) SERS spectra of R6G at different concentrations for original GNS4 probe. (**b**) SERS spectra of R6G at different concentrations for GNS4 probe with 3.5 minutes acetone treatment time.

Figure 6a shows the SERS spectra acquired when the original GNS4 probe was adsorbing R6G molecules with different concentrations. A detection limit of R6G up to 10^−9^ M was obtained. While using the optimal treatment condition (3.5 minutes immersion), a detection limit as low as 10^−11^ M was obtained by using GNS4 substrates as shown in Fig. 6b. It corresponds to a ~100 fold enhancement of the SERS sensitivity. This enormous improvement of the SERS sensitivity is attributed to the following two reasons. The first one is the Raman signal enhancement due to the distance decrease between the metal nanostructures and the analyte molecules, and the second one is the significant fluorescence inhibition of analyte molecules due to the direct schottky contact between metal and molecules. Because SERS signal is easily annihilated among strong background fluorescence, fluorescence inhibition could effectively contribute to the SERS signal trace detection. To verify the positive contribution of fluorescence inhibition on the SERS enhancement, we did a comparison experiment using adenine as an SERS analyte. Adenine is also a widely used SERS analyte, however, with a much weaker fluorescence compared with R6G. When such analytes are used, one can obtain the relationship between the SERS enhancement and the distance change between the analyte and the metal nanostructures without the influence of fluorescence. The SERS spectra of adenine molecules were obtained for original GNS4 probe at different concentrations as shown in Fig. S4(a). We measured a detection limit of R6G up to 10^−8^ M. While using the optimal treatment condition (3.5 minutes acetone immersion), a detection limit of adenine, as low as 10^−9^ M, was obtained as shown in Fig. S4(b). The enhanced multiple (~10 fold) of the sensitivity is an order less than that of R6G (~100 fold). This comparison experimental result clearly verified that the Raman signal enhancement due to the distance decrease between the metal nanostructures and the analyte molecules is not the only reason for analyte with strong fluorescence. The much better improvement of SERS sensitivity of R6G molecules is also attributed to the effective fluorescence inhibition after surface modification of GNSs. The above experimental results proved that our proposed surface modification method by acetone immersion of GNS substrate is an effective technique suitable for the improvement of SERS signal of various analyte, especially for trace detection of Raman analyte with strong fluorescence. We also studied the uniformity and time stability of the acetone-treated substrates using the SERS spectrum of R6G shown in Fig. S6. The results showed the good uniformity and the long-term stability of this SERS probe.

**Figure 7:**
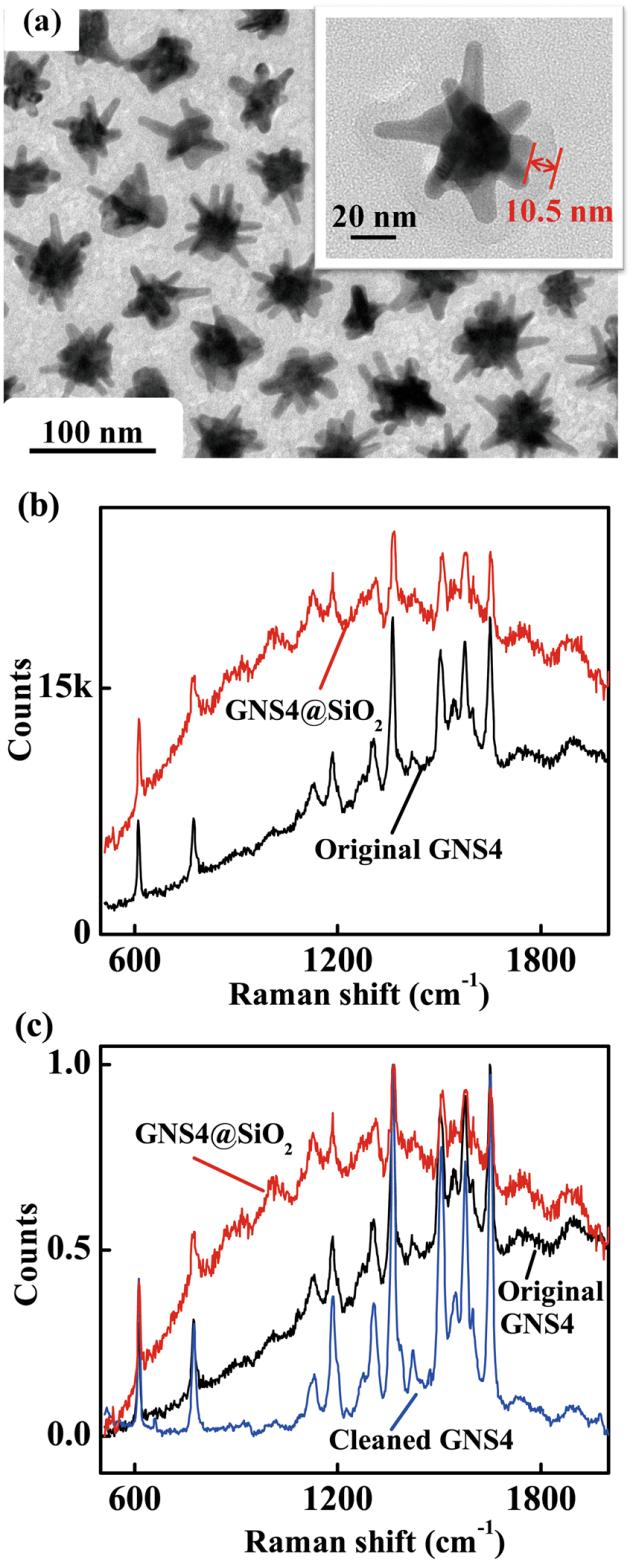
(**a**) TEM micrograph of the GNS4@SiO_2_ (inset: higher magnification TEM image for individual GNS4@SiO_2_). (**b**) SERS spectra of R6G at a concentration of 1 × 10^−7^ M for original GNS4 and GNS4@SiO_2_ probes. (**c**) normalized Raman spectra of the GNS4@SiO_2_ (red line), original GNS4 (blue line), and cleaned GNS4 (black line).

To further indicate that the increase of SERS EF arises from both the inhibition of fluorescence and the distance decrease between the metal nanostructures and the analyte molecules, we designed another experiment. It is well known that when the distance between metal nanostructure and fluorescence molecules increased, the fluorescence intensity would be increased by the LSPR of metal nanostructure^2^. Here SiO_2_ was used as an isolation layer to increase the space between the metal and R6G molecules (Fig. 7a). The thickness of SiO_2_ shell was about 10 nm (inset of Fig. 7a). After the immersion of 10^−7^ M R6G solution, we compared the SERS performance of original GNS4 and GNS4@SiO_2_ SERS substrates, as shown in Fig. 7b. As expected, the GNS4@SiO_2_ SERS substrates showed weakened SERS signals due to the strong fluorescence noises. We also compared these two samples with cleaned GNS4 SERS substrates. The normalized (dividing each group of data by their maximum values) Raman spectra (Fig. 7c) of the GNS4@SiO_2_, original GNS4, and cleaned GNS4 exhibit simultaneously existed Raman scattering enhancement and inhibiting fluorescence. It is clear that with the decrease of the thickness of the shell, fluorescence noises were inhibited while SERS signals enhanced. We also compared the adenine’s SERS performance of original GNS4, cleaned GNS4 and GNS4@SiO_2_ SERS probes, as shown in Fig. S5. The results were in accordance with that of R6G molecules.

**Figure 8:**
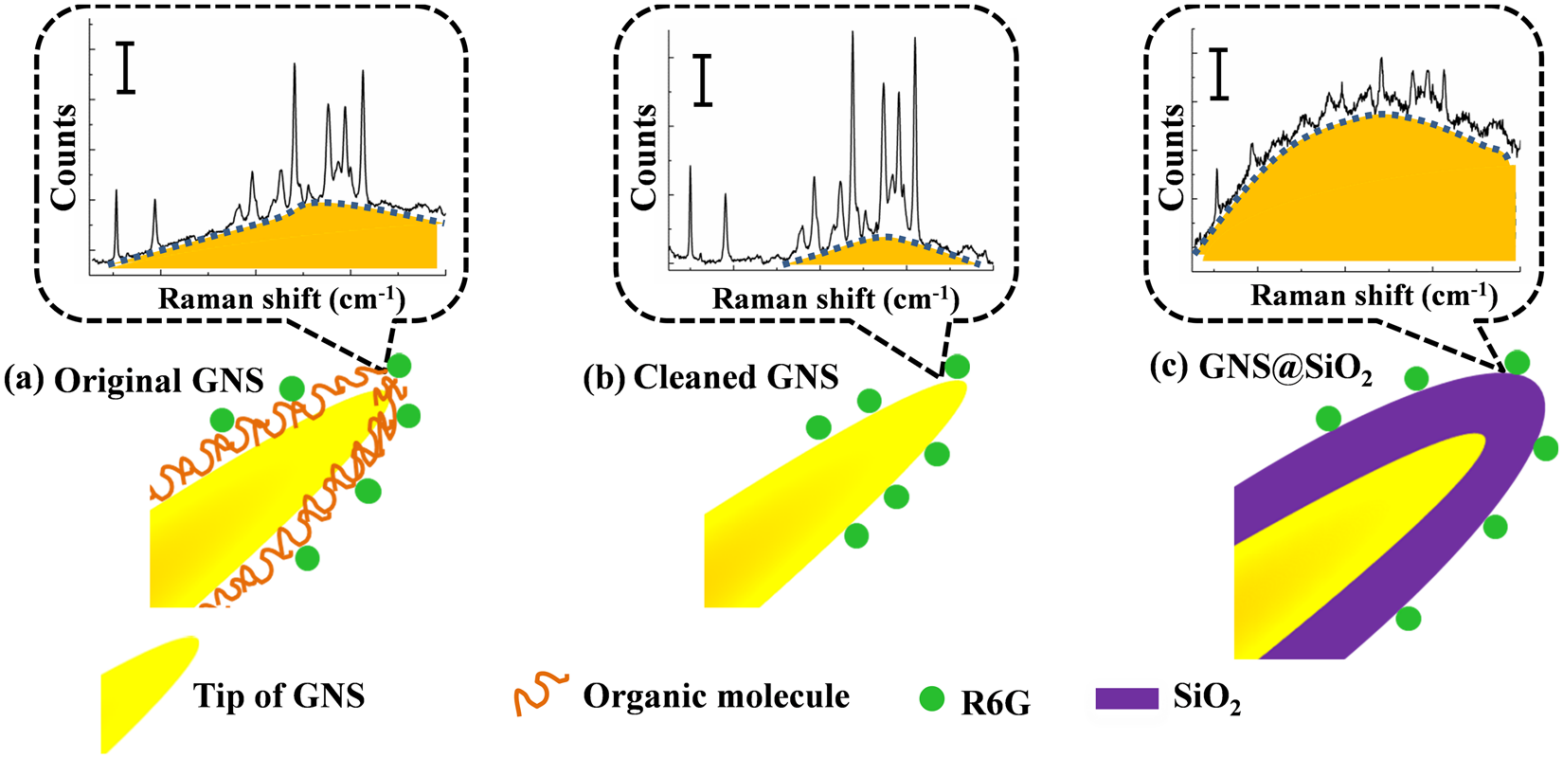
Schematic illustrations of surface-treatment GNS demonstrating the competing relationship of fluorescence and surface enhanced Raman scattering. (**a**) Original GNS. (**b**) Cleaned GNS. (**c**) GNS4@SiO_2_.

As shown in Fig. 8, our whole assumption processes were clearly exhibited by three comparison conceptual graphs. Figure 8a illustrated the original GNS probe with organic shell on the surface of GNSs, which will generate fluorescence noise during the SERS measurement process (the block diagram of Fig. 8a). There were no further post-processing to the metal nanoparticles before the SERS experiments, leading to negative effects in SERS detection in most literatures^4, 9, 12, 18–20^. After immersion treatment in organic solvents such as acetone, the organic shells of GNSs were dissolved at a suitable time and a cleaned GNSs SERS probe was obtained as shown in Fig. 8b. We clearly observed an enhanced SERS signal as well as an inhibition of fluorescence noise when the surface of metal and R6G molecules are closely contacted (the block diagram of Fig. 8b). We have experimentally proved that the effect of acetone is very special, which is slow, gradual and controllable in terms of treating the SERS probes. It could chemically tune the characteristics of the GNSs^37^. As shown in Fig. 8c, we designedly added an isolation layer of SiO_2_ to increase the distance between the metal and R6G molecules. It is an effective treatment method in fluorescence enhancement field, but has an extremely negative impact on the SERS detection (the block diagram of Fig. 8c). Thus, on the basis of a series of comparison experiments along with a visual representation, the relationship between Raman scatting and fluorescence of GNSs SERS probes were fully validated. Compared with other methods to remove the surface shells, such as electrochemical method^38^, plasma cleaning method^39^ and UV/ozonolysis method^40^, the current proposed acetone immersion method has advantages of being straightforward, fast, low-cost, and independent on instruments.

**Figure 9:**
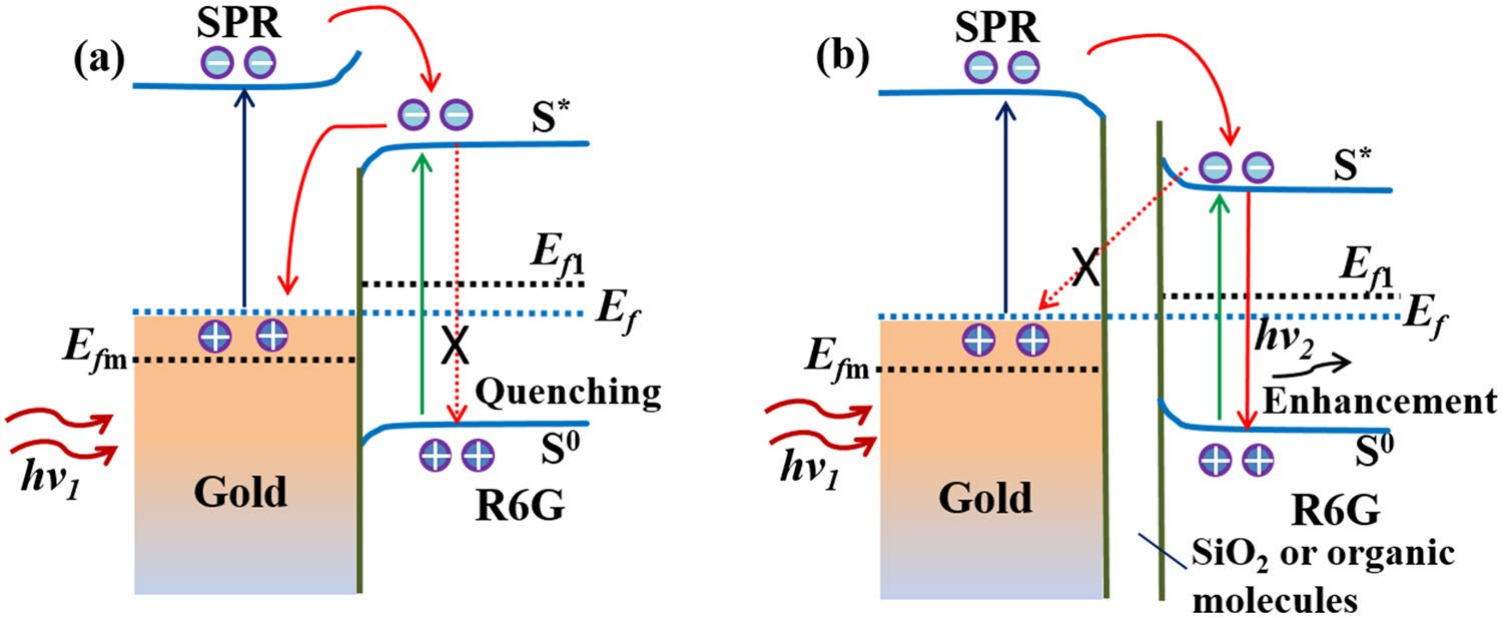
Schematic diagram of the surface resonance charge transfer. (**a**) The fluorescence dye R6G having close contact with Gold. (**b**) The fluorescence dye R6G and Gold was isolated by SiO_2_ or organic molecules. (Xmeans the drop process was restrained).

The relationship between Raman scattering and fluorescence has been experimentally demonstrated above. Then, we gave further explanation in principle using the plasmon-induced surface resonance energy transfer theory. This newly observed theory described the possible mechanism of the charge transfer enhanced by plasmon-induced hot electron generation at nanoparticle/SiO_2_ interface, which show potential applications in the development of new-concept optoelectronic devices^41^. When the GNS is directly contacted with the R6G (Fig. 9a), the hot electron on the surface of GNS has a higher energy than that of S^*^ (excited state) edge of R6G owning to the LSPR properties of metallic nanoparticles. These excited hot electrons will drop to the S^*^ of R6G by the SPR energy transfer. In addition, the R6G molecules itself absorbed a large number of photons, allowing the electrons transfer from the S^0^ (ground state) to S^*^(green line). Then, these electrons transfer to the surface of GNS, leading to fluorescence quenching phenomenon due to the non-radiative effect^42, 43^. In another case (Fig. 9b), organic molecules or SiO_2_ shells as an isolation layer block the hot electron transfer from S^*^ to the surface of GNS. The high-energy charges will transfer from the S^*^ to S^0^ directly, increasing the probability of recombination between the electrons and the holes. This charge transfer process will highly enhance the fluorescence radiation in R6G^44, 45^. This analysis above gives a reasonable explanation to the relationship between the thickness of shells and fluorescence noises observed in experiments.

## Conclusion

In summary, we have demonstrated the simultaneously existed Raman scattering enhancement and inhibiting fluorescence during SERS signal detection by a series of comparative experiments. The results indicated that the fluorescence phenomenon is arising from the analyte molecules, which is usually a serious problem in the SERS signal detection, can be greatly inhibited by using suitable surface modification technology. Meanwhile, various technologies including precise control of GNSs morphology, large-scale and uniform preparation of GNSs substrates were also studied. The intrinsic physical mechanisms of the fluorescence quenching effect and SERS enhancement arising from the surface plasmon assisted charge transfer process were systematically investigated. An optimized SERS signal sensitivity, as high as the level of 10^−11^ M, was achieved when R6G was used as the analyte. Finally, we obtained an optimum sensitivity of GNSs SERS probes which was 10^2^ times higher than that of the original SERS probe, which is a very competitive result for highly sensitive SERS detection. These newly observed physical mechanism and originally demonstrated technological methods may find promising applications in the fields of molecular spectroscopy analysis and nanotechnology.

## Methods

### Materials

Trisodium citrate (≥99.9%), Sodium silicate solution (27%), (3-Aminopropyl)trimethoxysilane (APTMS, 97%), ascorbic acid (AA, >99.5%) and silver nitrate (AgNO_3_, >99.9%) were purchased from Sigma-Aldrich. Gold chloride trihydrate (HAuCl_4_⋅3H_2_O, >99.9%), hydrochloric acid (HCl, >98.0%), Rhodamine 6G (R6G, >99.0%) were purchased from sinopharm chemical reagent Co. Ltd. Ultrapure water from Milli-Q (Millipore, America, resistivity >18.4 MΩ⋅cm) source was used throughout the experiments. All materials were used without further purification.

### Seeds synthesis

100 ml of 1 mM HAuCl_4_⋅3H_2_O solution were heated to boiling under vigorous stirring. Then 15 ml of 1% trisodium citrate solution was added. The reactant was cooled to stop reaction after 15 minutes boiling.

### GNSs synthesis

100 μL of 1 M HCl were added to 100 ml of 0.25 ml HAuCl_4_⋅3H_2_O in a 150 ml erlenmeyer flask at room temperature under stirring of 700 rpm. Then 1 ml of seed solution purified by a 0.22 μm nitrocellulose membrane filter was added. Then, 1 ml of AgNO_3_ solution with a suitable concentration and 500 μl of 100 mM AA solution were added simultaneously. The color of the solution changed rapidly from shiny red to blue. The reaction was completed within 30 s. By tuning the concentration of the AgNO_3_ solution, a series of GNSs samples with different morphologies were obtained.

### GNS@SiO_2_ synthesis

300 mL of GNS4 solution were centrifuged (4000 rpm, 30 minutes) and redispersed in 100 mL of water. 4 mL of APTMS (1 mM) were then added to dispersed solution and stirred for 15 minutes. Then, 30 mL of Sodium silicate solution (0.54 wt%) were added and stirred for 3 minutes. The reaction solution was heated for 2 h (100 °C, 600 rpm). After centrifuging the solution (4000 rpm, 30 minutes), the GNS4@SiO_2_ samples were obtained^46^.

### Characterization

The structural features of the GNSs were characterized using scanning electron microscope (SEM, Zeiss Ultra Plus) and transmission electronic microscopy (TEM; Fei Tecnai G20). Optical extinction spectra were measured using the fiber optic spectrometer (NOVA, Ideaoptics Technology Ltd., China). Raman spectra were obtained using the Laser Confocal Raman Micro-spectroscopy (LCRMS, LabRAM HR UV-Visible, France), where 50× long working distance objective lens was used. The laser power is 1.2 mW for 514 nm. The time of acquisition is 10 s. R6G were uniformly attached on the surface of GNSs by immersing SERS substrates into the R6G solution in different concentrations.

## Electronic supplementary material

SI
