# Supplementary material for: Investigation of simultaneously existed Raman scattering enhancement and inhibiting fluorescence using surface modified gold nanostars as SERS probes
Source: Sci Rep. 2017 Jul 28;7:6813. doi: 10.1038/s41598-017-07311-8 (PMC5533772; doi:10.1038/s41598-017-07311-8)
Supplement: Supplementary file 1 — SI [file 41598_2017_7311_MOESM1_ESM.doc]

Supporting Information of

**Investigation of** **simultaneously existed Raman scattering enhancement and inhibiting fluorescence using surface modified gold nanostars as SERS probes**

Feng Shan^1,2,3^,^†^, Xiao-Yang Zhang^1,2,3^,^†^, Xing-Chang Fu^1,3^, Li-Jiang Zhang^1,3^, Dan Su^2,3^, Shan-Jiang Wang^1,3^, Jing-Yuan Wu^1,3^, and Tong Zhang^1,2,3,*^

*^1^Joint International Research Laboratory of Information Display and Visualization, School of Electronic Science and Engineering, Southeast University, Nanjing, 210096, People’s Republic of China.*

*^2^Key Laboratory of Micro-Inertial Instrument and Advanced Navigation Technology, Ministry of Education, and School of Instrument Science and Engineering, Southeast University, Nanjing, 210096, People’s Republic of China.*

*^3^Suzhou Key Laboratory of Metal Nano-Optoelectronic Technology, Suzhou Research Institute of Southeast University, Suzhou, 215123, People’s Republic of China.*

*corresponding author email: [*tzhang@seu.edu.cn*](mailto:tzhang@seu.edu.cn).

^†^These authors contributed equally to this work.

***1*** ***Energy-dispersive X-ray (EDX) examinations of the GNSs.***

EDX spectrum was used to detect elements on the surface of GNS4 before and after immersing in the acetone solution. On the surface of GNS4, the EDX spectrum (Figure S1 (a)) confirms the existence of C, O, Cl and Ag signals, which come from the react reagent of ascorbic acid (C_6_H_8_O_6_), hydrochloric acid (HCl) and silver nitrate (AgNO_3_). However, after 3.5 minutes of immersing in acetone, the C, O and Cl signals disappeared as shown in Figure S1 (b). Therefore, EDX spectrum analysis proves that the organic molecules layer on the surface of GNS4 could be dissolved by acetone.


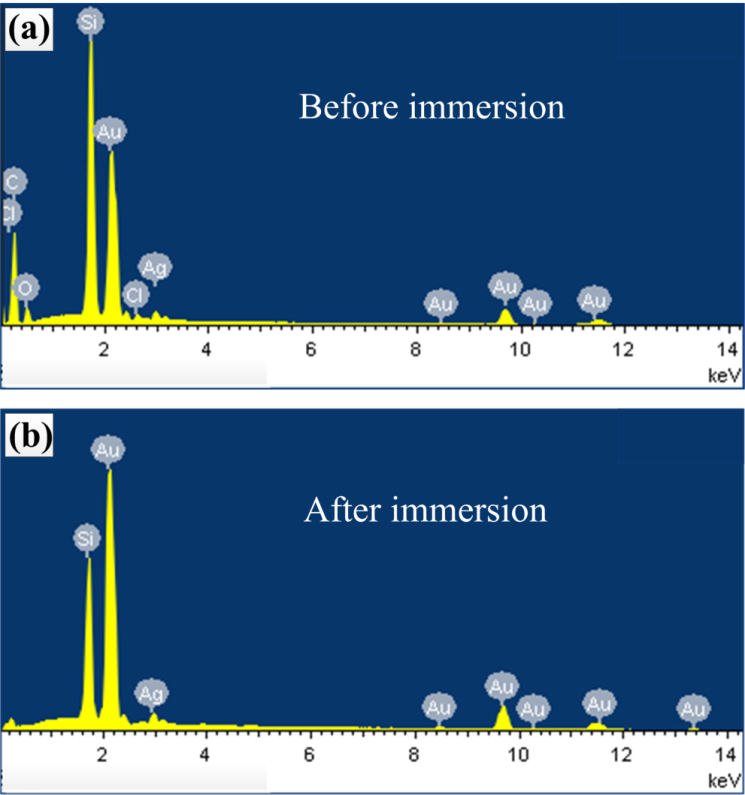


**Figure S1**. EDX spectra on the surface of GNS4 (a) before and (b) after 3.5 minutes immersion in acetone solution.

***2 Investigation of GNS4 and GNS4 films with surface modification by acetone immersion.***

To visually show the effect of the acetone immersion, three types of samples were produced including the original GNS4, the GNS4 immersing in acetone for 3.5 minutes and the GNS4 immersing in acetone for 5 minutes. Transmission electron microscopy (TEM) images of these three samples are shown in Fig. S2. Compared with original GNS4 (Fig.S2(a)), the morphology of CNS4 immersing in acetone for 3.5 minutes (Fig.2(b)) did not change obviously. Meantime, the organic molecules layer on the surface of GNS4 have been dissolved effectively by acetone, as shown in EDX spectrum of Fig. S1. It is therefore corresponds to the best SERS probes which directly contact with R6G analyte and keep their sharp tips, ie. hot spots supporter. However, once the immersion time of acetone is further increased, the GNS4 probes gradually decomposed without the protection of organic shells. It can be seen clearly from TEM image of GNS4 after 5 minutes acetone immersion as shown in Fig. S2(c). In this case, the SERS signal decreased because the ‘hot spots’ in the GNS4 tips were no longer existed after long time immersion in acetone. The extinction spectra of GNS4 film with different immersion time in acetone in Fig. S2(d) further prove this explanation. A obvious blue-shift of the main extinction band was observed from the extinction spectra when the immersion time was increased due to the removal of organic shells and the decomposition of tips of GNS4 by acetone solvent. The result is in accordance to the analysis of TEM images.


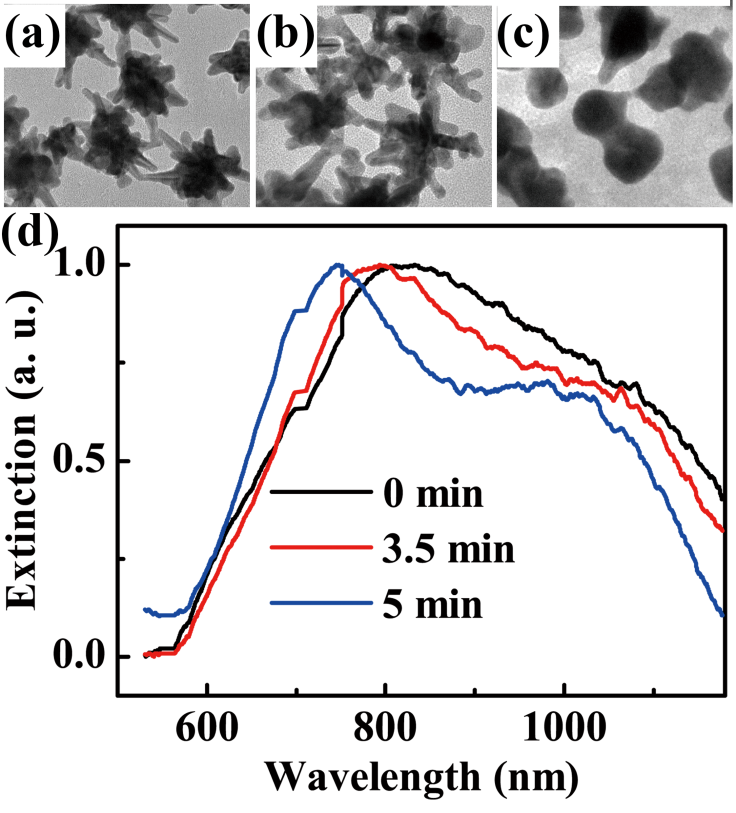


Figure S2. Comparison of SEM images of GNS4 (a) original, (b) after 3.5 minutes immersion and (c) after 5 minutes immersion. (d) Extinction spectra for the films of original GNS4 (black curve), after 3.5 minutes immersion (red curve) and after 5 minutes immersion (blue curve).

***3 Raman spectra of R6G on ITO glass substrate.***

The Raman spectra of R6G at a concentration of 1×10^-2^ M were measured on ITO glass substrate as shown Fig. S3. We observed a strong fluorescence background clearly in the Raman spectrum. It would overwhelm the Raman signal of R6G. Therefore, in order to improve the SERS signal, the inhibition of fluorescence is critical.


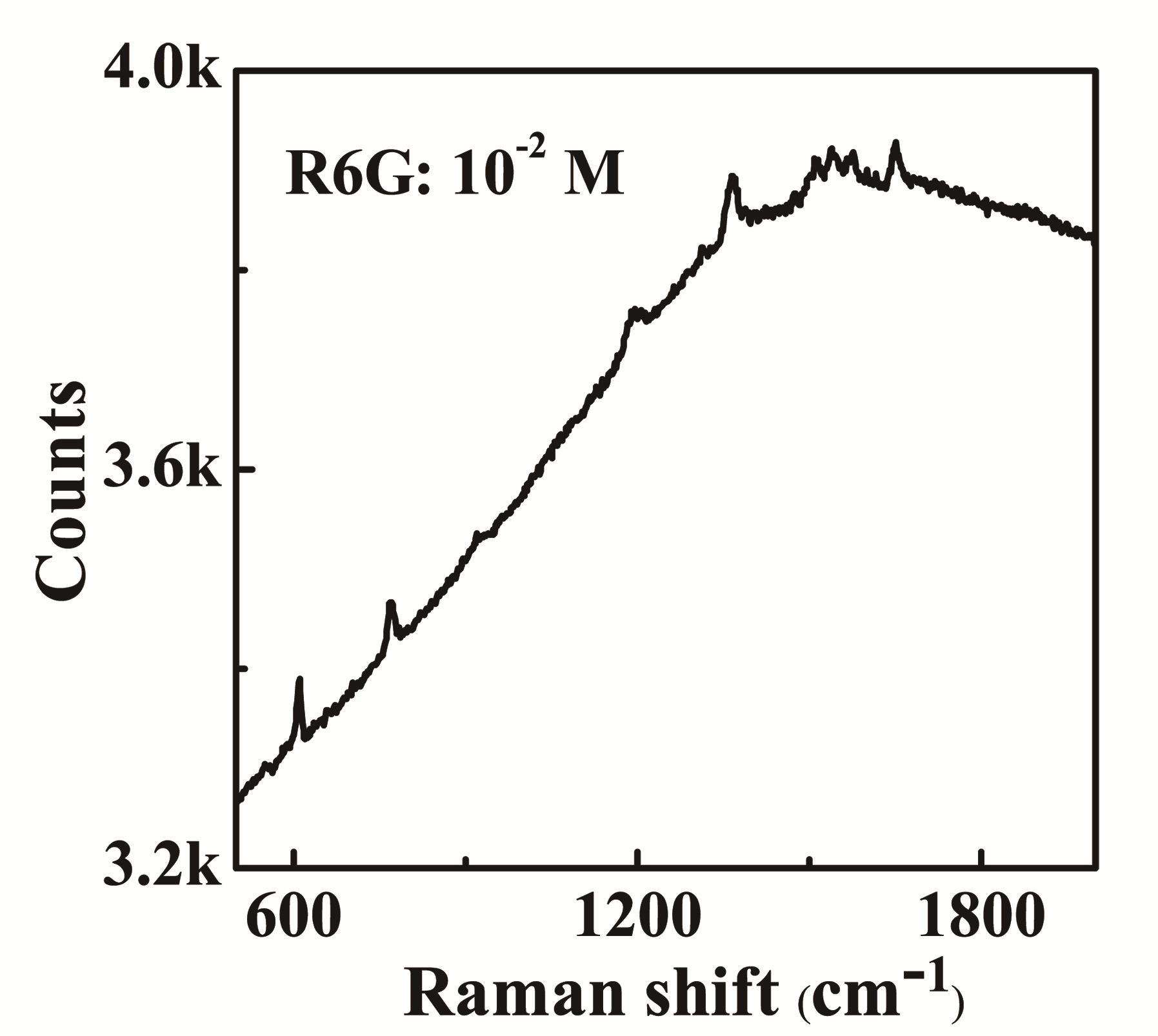


Figure S3. Raman spectra of R6G at a concentration of 1×10^-2^ M under ITO glass substrate.

***4 SERS spectra of adenine molecules for GNS4 probe before and after acetone immersion.***

Fig. S4(a) shows the SERS spectra acquired when the original GNS4 probe was adsorbing adenine molecules of different concentrations. A detection limit of adenine down to 10^-8^ M was obtained. While using the optimal treatment condition (3.5 minutes immersion), a detection limit of adenine, as low as 10^-9^ M, was obtained by using cleaned GNS4 substrates as shown in Fig. S4(b). We obtained a ~10 fold enhancement of the SERS sensitivity after immersing the SERS substrates into acetone solution for 3.5 minutes. The enhanced multiple of the sensitivity is less than that of R6G (~100 fold). We also analyzed the reason why the SERS signal enhancement of R6G is greater than that of adenine. Because the analyte with a stronger fluorescence overwhelms SERS signal to a larger extent. Once the fluorescence was inhibited, their SERS signals would get a greater increase. It is proved that this strategy can inhibit the fluorescence and improve the SERS signal intensity.


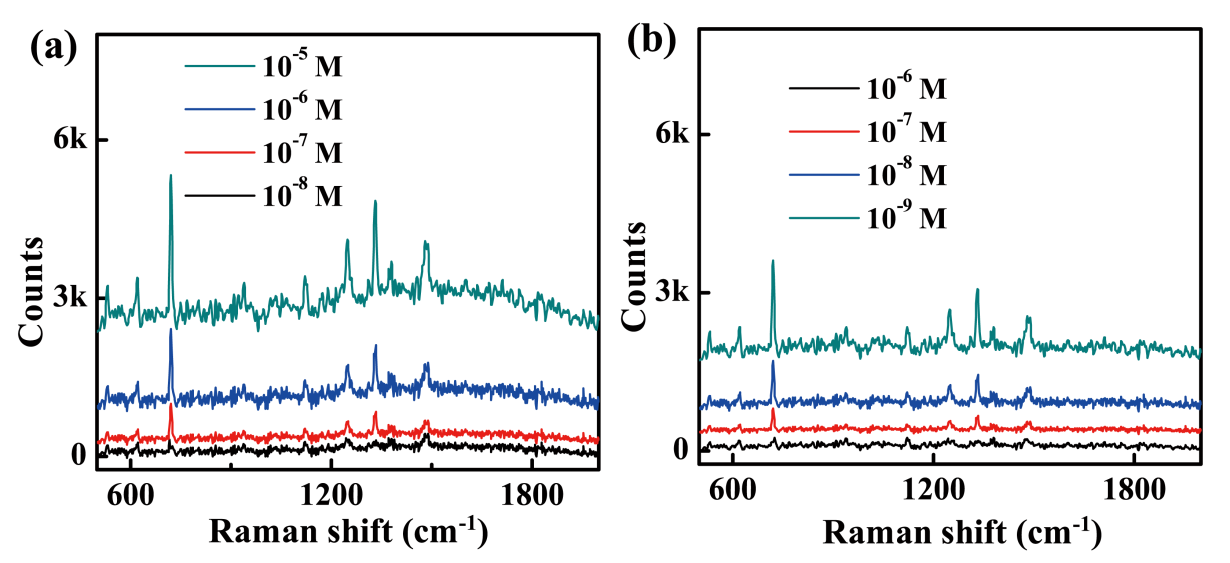


Figure S4. SERS spectra of adenine molecules at different concentration for original GNS4 probe. (b) SERS spectra of adenine molecules at different concentration for GNS4 probe at 3.5 minutes acetone treatment time.

***5 SERS spectra of adenine molecules for original GNS4, cleaned GNS4 and GNS4@SiO_2_ probes.***

After the immersion of 10^-5^ M adenine solution, we compared the SERS performance of original GNS4, cleaned GNS4 and GNS4@SiO_2_ SERS substrates, as shown in Fig. S5. As expected, the GNS4@SiO_2_ SERS substrates showed fluorescence noises which dramatically overwhelmed the Raman signals. It is clear that with the decrease of the thickness of the shell, fluorescence noises were inhibited while SERS signals were enhanced. The results were in accordance with that of R6G molecules.


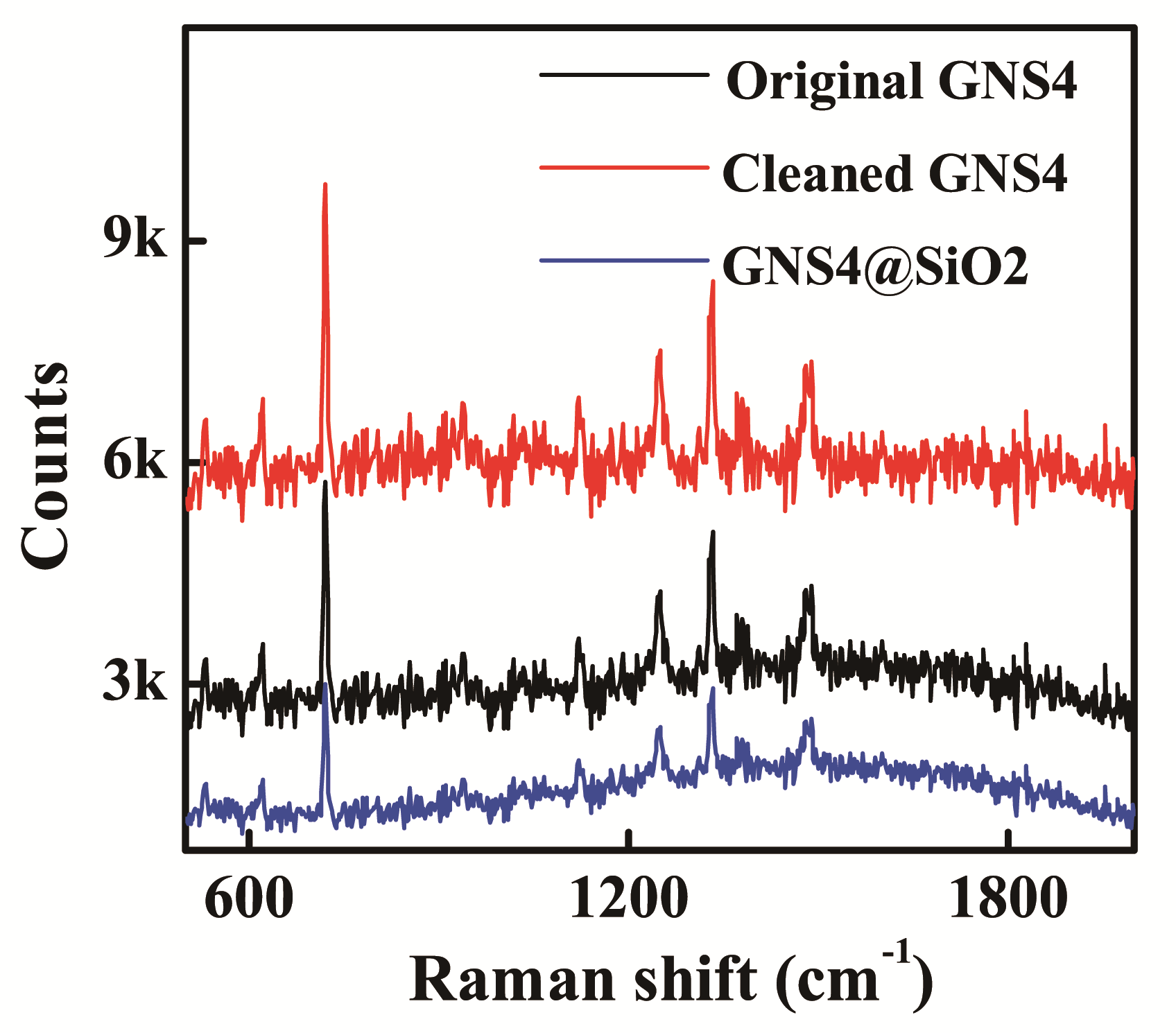


Figure S5. SERS spectra of adenine molecules at a concentration of 1×10^-5^ M for original GNS4, cleaned GNS4 and GNS4@SiO_2_ probes.

***6 The demonstration of SERS spectrum’s uniformity and time stability on the cleaned GNS4 substrates.***

We also examined the uniformity and time stability of the acetone-treated substrates using the SERS spectrum of R6G shown in Fig. S6. Ten points on the acetone treated GNSs SERS probes were selected randomly. It is shown that the uniformity of this SERS probe is very good. Meanwhile, we found that the SERS spectrum of R6G almost did not change after 15 days. As is known, the gold itself is very stable. Therefore, GNSs SERS substrates are promising candidates for high-sensitive SERS applications.


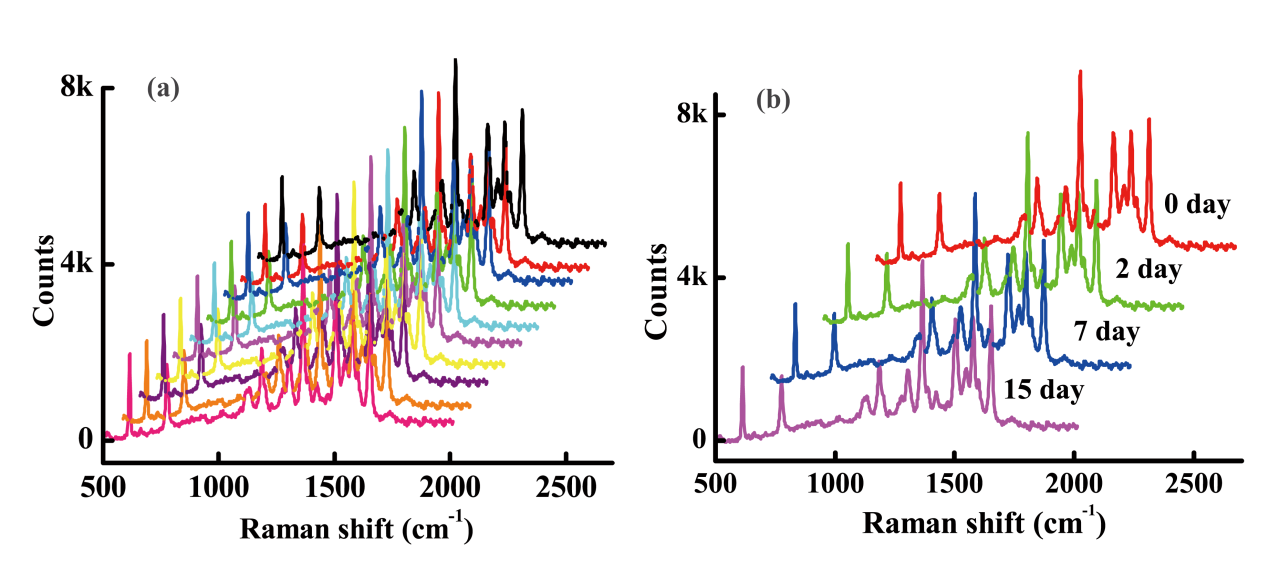


Figure S6. (a) R6G SERS spectra of ten random points on cleaned GNS4 with a concentration of 1×10^-7^ M. (b) R6G SERS spectra of different intervals on cleaned GNS4 with a concentration of 1×10^-7^ M.
